# Crystal structure of (*E*)-1,2-bis­(6-bromo-9-hexyl-9*H*-carbazol-3-yl)ethene

**DOI:** 10.1107/S2056989018002098

**Published:** 2018-02-13

**Authors:** Ying Feng, Wei Guo, Zhi Liu, Xinyu Luo, Dingchao Zhang, Li Li, Deliang Cui

**Affiliations:** a State Key Laboratory of Crystal Materials, Shandong University, Jinan 250100, Shandong Province, People’s Republic of China

**Keywords:** π-conjugated, carbazole derivative, π–π and C—H⋯π inter­molecular inter­actions, C—H⋯Br short contacts., crystal structure

## Abstract

The title compound, C_38_H_40_Br_2_N_2_, crystallizes in the triclinic space group *P*-1 with two mol­ecules in a unit cell. The two carbazole groups are nearly coplanar, making a dihedral angle of 16.90 (5)°, and are bridged by vinyl. The crystal structure features π–π and C—H⋯π inter­actions and C—H⋯Br short contacts.

## Abstract 2

In the title compound, the two carbazole groups are nearly coplanar, making a dihedral angle of 16.90 (5)°, and are bridged by vinyl. The crystal structure features π–π and C—H⋯π inter­actions and C—H⋯Br short contacts.

## Chemical context   

To date, π-conjugated organic mol­ecules have attracted considerable attention because of their applications in many fields, such as non-linear optics (Kim *et al.*, 2016 ▸; Percino *et al.*, 2016 ▸; Xue *et al.*, 2014 ▸) and optoeletronic devices (Shi *et al.*, 2016 ▸; Zhang *et al.*, 2015 ▸). Carbazole-based π-conjugated compounds have been utilized as the light-emitting layers in OLEDs (Liu *et al.*, 2006 ▸, 2014 ▸). The design of the title mol­ecule combines the advantages of several factors. Firstly, vinyl has been introduced to bridge mol­ecules; this is of importance for extension of the π-conjugated system, which is beneficial for carrier mobility (Wang *et al.*, 2012 ▸). Secondly, introducing long alkyl substituents to carbazole cores is an effective method to solve poor solubility (Teetsov & Fox, 1999 ▸) and fluorescence quenching in the solid state (Hua *et al.*, 2015 ▸). In addition, introduction of Br into the structure of vinyl-bridged carbazoles can enhance inter­molecular inter­actions by forming non-classical hydrogen bonds. Br-substituted mol­ecules are excellent inter­mediate products since the bonding energy of the C—Br bond is weaker than that of C—H, and Br substituents are easily replaced by other substituents.
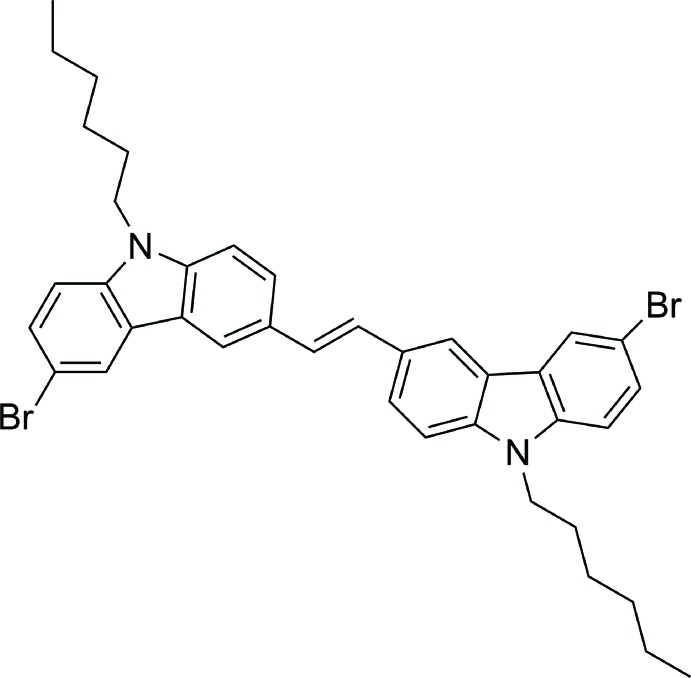


## Structural commentary   

**Figure 1:**
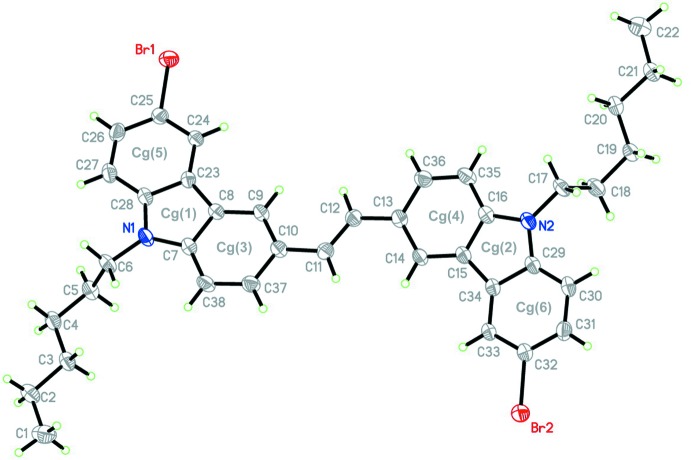
The mol­ecular structure of the title compound, **1**, with the atom labelling. Displacement ellipsoids are drawn at the 30% probability level.

The title compound crystallizes in the space group *P*_ī_ with one mol­ecule in the asymmetric unit, as shown in Fig. 1 ▸. The mol­ecule is an (*E*) isomer and has approximate *C*_s_ symmetry. The mean deviation from the plane of the cabazole unit including N1 is 0.0272 Å, with deviations of 0.159 (2) Å for C11 and 0.059 (2) Å for Br1, while the mean of the cabazole unit including N2 is 0.0224 Å with deviations of 0.052 (2) Å for C12 and 0.084 (2) Å for Br2. Note that there is a double bond between carbon atoms C11 and C12. Each carbazole group is planar, excluding hexyl groups, and its respective peripheral atoms such as bromine and the double-bonded carbon atoms were accommodated in a planar geometry, as shown by the C6—N1—N2—C17 torsion angle of −147.5 (2)° and the Br1—C25—C32—Br2 torsion angle of −167.70 (3)°. The two carbazole groups are almost in the same plane, making a dihedral angle of 16.9 (5)°. The angles between the least-squares planes of neighboring rings are in the range of 1.00–1.42°. Furthermore, they are *trans* to the C=C double bond, as indicated by the C10—C11—C12—C13 torsion angle of 176.1 (2)°. The intra­molecular Br1⋯Br2 distance of 16.710 (5) Å is much longer than the sum of the van der Waals radii (3.7 Å) and the angle between C—Br bonds is 169.4°, indicating that the title mol­ecule forms an extended, conjugated π-system.

## Supra­molecular features   

**Figure 2:**
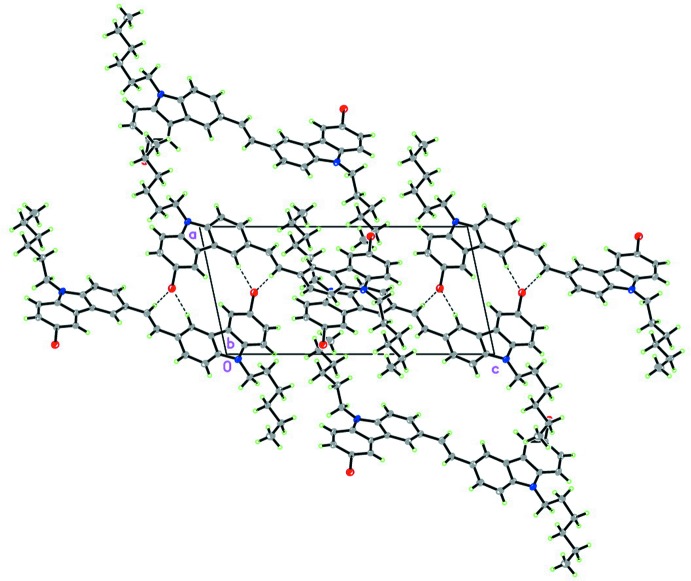
The crystal packing of the title compound **1** viewed along the *b* axis. Details of C—H⋯Br also were showed.

In the crystal, the mol­ecules stack in a face-to-face manner along the *b* axis (see Fig. 2 ▸). Adjacent mol­ecules are staggered and inter­locked through their aromatic units, which assume face-to-face orientations. The distances and angles between them indicate the presence of well-defined inter­molecular π–π inter­actions (Hunter *et al.*, 1990 ▸) [*Cg*1⋯*Cg*5(1 − *x*, 2 − *y*, 1 − *z*) = 3.6898 (13) and *Cg*2⋯*Cg*6(−*x*, 1 − *y*, 2 − *z)* = 3.5000 (13) Å; *Cg*1, *Cg*2, *Cg*5 and *Cg*6 are the centroids of the N1/C7/C8/C23/C28, N2/C16/C15/C34/C29, C23–C28 and C29–C34 rings, respectively]. There are C—H⋯π inter­actions (Table 1 ▸) between neighboring mol­ecules along the *a* axis while weak C—H⋯Br short contacts link the mol­ecules into a chain-like arrangement in the *ac* plane (Table 1 ▸).

## Database survey   

A search of the Cambridge Crystallographic Database (WebCSD, Version 1.1.2, last update November 2016; Groom *et al.*, 2016 ▸) for (*E*)-1,2-di(9*H*-carbazol-3-yl)ethene, reveals six structures. The structure of (*E*)-1,2-bis­(9-hexyl-9*H*-carbazol-3-yl)ethene was determined successfully by our research group (Shi, Liu, Dong *et al.*, 2012 ▸; Shi, Liu, Guo *et al.*, 2012 ▸) and we have also investigated the propeller-shaped structures of two ethene derivatives substituted by carbazole, phenyl and dimesitylboron (Shi *et al.*, 2016 ▸). The single crystal structure of the ethene substituted by two cabazole groups and two phenyl rings has been reported (Liu *et al.*, 2014 ▸) as well as structures where the two carbazole groups are linked *via* several organic groups, including vinyl (Kumar *et al.*, 2006 ▸; Song *et al.*, 2008 ▸).

## Synthesis and crystallization   

All reactants and solvents were purchased and used without further purification. THF was dried by using Na in the presence of benzo­phenone and DMF was dried by using mol­ecular sieves. 9-Hexyl-9*H*-carbazole (**4**), 9-hexyl-9-carbazole-3-carbaldehyde (**3**) and 9-hexyl-9-carbazole-3-Br-6-carbaldehyde (**2**) were synthesized according to methods reported by our research group (Chen *et al.*, 2017 ▸; Shi, Liu *et al.*, 2012 ▸ ▸; Shi, Xin *et al.*, 2012 ▸ ▸).

**Figure 3:**
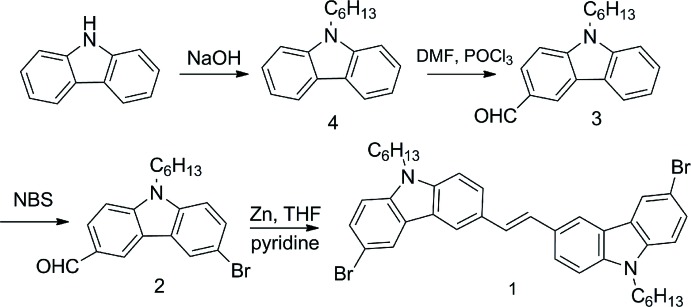
Reaction scheme.

The title compound **1** was synthesized through a McMurry reaction (see Fig. 3 ▸). (*E*)-1,2-Bis(6-bromo-9-hexyl-9*H*-carbazol-3-yl)ethene (**1**): Zn power (5.840 g, 80.0 mmol) was mixed with THF (200.0 mL) and stirred sharply on the flask under Ar. Pure di­chloro­methane (30.0 mL) was poured into a constant pressure funnel and then TiCl_4_ (4.42 mL, 40.0 mmol) was injected into the di­chloro­methane. The mixture was added dropwise to the flask. The reaction system was heated at 353 K and stirred for 3 h. After cooling to room temperature, compound **2** was dissolved in THF (100.0 mL), added dropwise to the flask for 2 h at 273 K, then heated to 353 K and stirred for 24 h. Finally, the mixture was poured into saturated NaHCO_3_ solution and stirred sharply for 3 h. The reaction solution was extracted with di­chloro­methane. The solvent was washed with deionized water and saturated brine three times, then dried with anhydrous magnesium sulfate. After the solvent had been removed under reduced pressure, the residue was purified by flash chromatography on silica gel using di­chloro­methane–petroleum ether (1: 4 *v*:*v*) as eluent to achieve a yellow solid. Pale-yellow block-shaped crystals were obtained by recrystallization from the mixed solvent *n*-hex­ane/methyl­ene chloride (0.878 g). Yield: 64.3%.

^1^H NMR (300 MHz, CDCl_3_, 298 K, TMS): *δ* = 8.24 (*d*, *J* = 1.8 Hz, 2H; Ar-H), 8.19 (*d*, *J* = 1.5 Hz, 2H; Ar-H), 7.74 (*d*, *J* = 1.8 Hz, 2H; Ar-H), 7.71 (*d*, *J* = 1.5 Hz, 2H; Ar-H), 7.55 (*dd*, *J* = 1.8 Hz, 2H; Ar-H), 7.52 (*d*, *J* = 2.4 Hz, 2H; Ar-H), 7.40 (*s*, 1H; Ar-H), 7.38 (*s*, 1H; Ar-H), 4.27 (*t*, *J* = 7.5 Hz 4H; hexyl-H), 1.91–1.81 (*m*, 4H, hexyl-H), 1.42–1.45 (*m*, 12H; hexyl-H), 0.87 ppm (*t*, *J* = 7.0 Hz, 6H; hexyl-H); ^13^C NMR (75 MHz, CDCl_3_, 298 K, TMS): *δ* = 139.73, 138.97, 129.01, 127.85, 126.61, 124.46, 124.12, 122.68, 121.73, 117.87, 111.18, 109.78, 108.67, 42.85, 31.05, 28.44, 26.44, 22.04, 13.51 ppm; FTIR: 3030, 2955, 2944, 2926, 2864, 1839, 1736, 1628, 1596, 1488, 1465, 1450, 1383, 1349, 1302, 1286, 1244, 1220, 1194, 1152, 1134, 1053, 1019, 896, 867, 804, 790, 746, 730 cm^−1^; HRMS (MALDI–TOF): *m*/*z*: calculated for C_38_H_40_Br_2_N_2_: 682.2; found: 683.7.

## Refinement   

Crystal data, data collection and structure refinement details are summarized in Table 2 ▸. C-bound H atoms were refined using a riding model with C—H = 0.93–0.97 Å and *U*_iso_(H) = 1.2–1.5*U*_eq_(C).

## Supplementary Material

Crystal structure: contains datablock(s) I. DOI: 10.1107/S2056989018002098/ex2004sup1.cif

Structure factors: contains datablock(s) I. DOI: 10.1107/S2056989018002098/ex2004Isup2.hkl

Supporting information file. DOI: 10.1107/S2056989018002098/ex2004Isup3.cml

CCDC reference: 1821846

Additional supporting information:  crystallographic information; 3D view; checkCIF report

## Figures and Tables

**Table 1:** Hydrogen-bond geometry (Å, °) *Cg*6 is the centroid of the C29–C34 ring.

*D*—H⋯*A*	*D*—H	H⋯*A*	*D*⋯*A*	*D*—H⋯*A*
C9—H9⋯Br1^i^	0.93	3.04	3.9062 (19)	157
C12—H12⋯Br1^i^	0.93	3.00	3.921 (2)	172
C11—H11⋯Br2^ii^	0.93	3.03	3.932 (2)	163
C14—H14⋯Br2^ii^	0.93	2.94	3.821 (2)	159
C21—H21*B*⋯*Cg*6^iii^	0.93	2.89	3.791 (3)	154

Symmetry codes: (i) 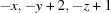; (ii) 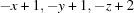; (iii) 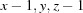.

**Table 2:** Experimental details

Crystal data	
Chemical formula	C_38_H_40_Br_2_N_2_
*M*_r_	684.54
Crystal system, space group	Triclinic, *P* 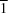
Temperature (K)	296
*a*, *b*, *c* (Å)	8.5553 (12), 11.4379 (16), 17.333 (2)
α, β, γ (°)	101.247 (2), 98.392 (1), 104.990 (2)
*V* (Å^3^)	1572.0 (4)
*Z*	2
Radiation type	Mo *K*α
μ (mm^−1^)	2.61
Crystal size (mm)	0.50 × 0.24 × 0.16

Data collection	
Diffractometer	Bruker APEXII CCD
Absorption correction	Multi-scan (*APEX2*; Bruker, 2005 ▸)
*T*_min_, *T*_max_	0.477, 0.659
No. of measured, independent and observed [*I* > 2σ(*I*)] reflections	18097, 7063, 5602
*R*_int_	0.036
(sin θ/λ)_max_ (Å^−1^)	0.649

Refinement	
*R*[*F* ^2^ > 2σ(*F* ^2^)], *wR*(*F* ^2^), *S*	0.031, 0.106, 0.77
No. of reflections	7063
No. of parameters	381
H-atom treatment	H atoms treated by a mixture of independent and constrained refinement
Δρ_max_, Δρ_min_ (e Å^−3^)	0.64, −0.37

Computer programs: *APEX2* (Bruker, 2005 ▸), *SAINT* (Bruker, 2005 ▸), *SHELXS97* (Sheldrick, 2008 ▸), *SHELXL97* (Sheldrick, 2008 ▸), *SHELXTL* (Bruker, 2005 ▸).

## supplementary crystallographic information

### Crystal data

**Table:**

C_38_H_40_Br_2_N_2_	*Z* = 2
*M**_r_* = 684.54	*F*(000) = 704
Triclinic, *P*1	*D*_x_ = 1.446 Mg m^−^^3^
*a* = 8.5553 (12) Å	Mo *K*α radiation, λ = 0.71073 Å
*b* = 11.4379 (16) Å	Cell parameters from 7151 reflections
*c* = 17.333 (2) Å	θ = 2.5–27.4°
α = 101.247 (2)°	µ = 2.61 mm^−^^1^
β = 98.392 (1)°	*T* = 296 K
γ = 104.990 (2)°	Block, pale yellow
*V* = 1572.0 (4) Å^3^	0.50 × 0.24 × 0.16 mm

### Data collection

**Table:**

Bruker APEXII CCD diffractometer	7063 independent reflections
Radiation source: fine-focus sealed tube	5602 reflections with *I* > 2σ(*I*)
Graphite monochromator	*R*_int_ = 0.036
φ and ω scans	θ_max_ = 27.5°, θ_min_ = 1.2°
Absorption correction: multi-scan (APEX2; Bruker, 2005)	*h* = −11→11
*T*_min_ = 0.477, *T*_max_ = 0.659	*k* = −14→14
18097 measured reflections	*l* = −22→22

### Refinement

**Table:**

Refinement on *F*^2^	Primary atom site location: structure-invariant direct methods
Least-squares matrix: full	Secondary atom site location: difference Fourier map
*R*[*F*^2^ > 2σ(*F*^2^)] = 0.031	Hydrogen site location: inferred from neighbouring sites
*wR*(*F*^2^) = 0.106	H atoms treated by a mixture of independent and constrained refinement
*S* = 0.77	*w* = 1/[σ^2^(*F*_o_^2^) + (0.1*P*)^2^] where *P* = (*F*_o_^2^ + 2*F*_c_^2^)/3
7063 reflections	(Δ/σ)_max_ = 0.033
381 parameters	Δρ_max_ = 0.64 e Å^−^^3^
0 restraints	Δρ_min_ = −0.37 e Å^−^^3^

### Special details

**Table:**

Geometry. All esds (except the esd in the dihedral angle between two l.s. planes) are estimated using the full covariance matrix. The cell esds are taken into account individually in the estimation of esds in distances, angles and torsion angles; correlations between esds in cell parameters are only used when they are defined by crystal symmetry. An approximate (isotropic) treatment of cell esds is used for estimating esds involving l.s. planes.
Refinement. Refinement of F^2^ against ALL reflections. The weighted R-factor wR and goodness of fit S are based on F^2^, conventional R-factors R are based on F, with F set to zero for negative F^2^. The threshold expression of F^2^ > 2sigma(F^2^) is used only for calculating R-factors(gt) etc. and is not relevant to the choice of reflections for refinement. R-factors based on F^2^ are statistically about twice as large as those based on F, and R- factors based on ALL data will be even larger.

### Fractional atomic coordinates and isotropic or equivalent isotropic displacement parameters (Å^2^)

**Table:**

	*x*	*y*	*z*	*U*_iso_*/*U*_eq_	
Br1	0.07518 (3)	1.08100 (2)	0.368357 (13)	0.03674 (9)	
Br2	0.48276 (3)	0.47768 (2)	1.151520 (13)	0.03649 (9)	
N1	0.4919 (2)	0.74884 (16)	0.43613 (10)	0.0292 (4)	
N2	−0.0392 (2)	0.70603 (16)	1.03984 (10)	0.0290 (4)	
C1	1.1045 (3)	0.4685 (2)	0.39475 (17)	0.0485 (6)	
H1A	1.1716	0.5189	0.4456	0.073*	
H1B	1.1708	0.4298	0.3643	0.073*	
H1C	1.0153	0.4052	0.4035	0.073*	
C2	1.0354 (3)	0.5495 (2)	0.34896 (14)	0.0357 (5)	
H2A	0.9748	0.4989	0.2961	0.043*	
H2B	1.1265	0.6142	0.3415	0.043*	
C3	0.9216 (3)	0.6105 (2)	0.39002 (13)	0.0316 (5)	
H3A	0.9814	0.6600	0.4432	0.038*	
H3B	0.8292	0.5459	0.3966	0.038*	
C4	0.8552 (3)	0.6937 (2)	0.34368 (13)	0.0311 (4)	
H4A	0.7862	0.6423	0.2927	0.037*	
H4B	0.9476	0.7526	0.3322	0.037*	
C5	0.7551 (3)	0.7660 (2)	0.38762 (13)	0.0320 (5)	
H5A	0.7431	0.8322	0.3619	0.038*	
H5B	0.8151	0.8044	0.4426	0.038*	
C6	0.5841 (3)	0.68408 (19)	0.38851 (13)	0.0319 (5)	
H6A	0.5210	0.6516	0.3337	0.038*	
H6B	0.5961	0.6136	0.4096	0.038*	
C7	0.4684 (2)	0.73873 (18)	0.51204 (12)	0.0260 (4)	
C8	0.3640 (2)	0.80997 (17)	0.53622 (12)	0.0240 (4)	
C9	0.3180 (2)	0.81224 (17)	0.61012 (11)	0.0264 (4)	
H9	0.2492	0.8592	0.6261	0.032*	
C10	0.3754 (2)	0.74392 (18)	0.66009 (12)	0.0274 (4)	
C11	0.3229 (3)	0.7348 (2)	0.73609 (12)	0.0298 (4)	
H11	0.3830	0.7018	0.7707	0.036*	
C12	0.1974 (3)	0.76918 (19)	0.76010 (12)	0.0290 (4)	
H12	0.1424	0.8068	0.7267	0.035*	
C13	0.1359 (2)	0.75442 (19)	0.83351 (12)	0.0283 (4)	
C14	0.2124 (2)	0.70651 (17)	0.89176 (11)	0.0267 (4)	
H14	0.3077	0.6841	0.8855	0.032*	
C15	0.1458 (2)	0.69252 (18)	0.95890 (12)	0.0265 (4)	
C16	0.0028 (2)	0.72944 (19)	0.96916 (12)	0.0278 (4)	
C17	−0.1801 (2)	0.7281 (2)	1.07101 (13)	0.0314 (5)	
H17A	−0.2301	0.6583	1.0923	0.038*	
H17B	−0.2617	0.7320	1.0272	0.038*	
C18	−0.1347 (3)	0.8482 (2)	1.13693 (14)	0.0361 (5)	
H18A	−0.0536	0.8443	1.1810	0.043*	
H18B	−0.0844	0.9182	1.1158	0.043*	
C19	−0.2842 (3)	0.8703 (2)	1.16877 (13)	0.0344 (5)	
H19A	−0.3470	0.7934	1.1792	0.041*	
H19B	−0.2453	0.9325	1.2196	0.041*	
C20	−0.3993 (3)	0.9134 (2)	1.11272 (12)	0.0306 (4)	
H20A	−0.3361	0.9880	1.0998	0.037*	
H20B	−0.4442	0.8492	1.0630	0.037*	
C21	−0.5411 (3)	0.9413 (2)	1.14864 (13)	0.0323 (5)	
H21A	−0.4966	0.9951	1.2023	0.039*	
H21B	−0.6138	0.8638	1.1537	0.039*	
C22	−0.6416 (3)	1.0029 (2)	1.10040 (16)	0.0448 (6)	
H22A	−0.5721	1.0817	1.0972	0.067*	
H22B	−0.7299	1.0161	1.1261	0.067*	
H22C	−0.6870	0.9502	1.0472	0.067*	
C23	0.3243 (2)	0.86664 (18)	0.47177 (11)	0.0241 (4)	
C24	0.2259 (2)	0.94360 (18)	0.45978 (12)	0.0244 (4)	
H24	0.1709	0.9708	0.4989	0.029*	
C25	0.2127 (2)	0.97824 (19)	0.38768 (12)	0.0276 (4)	
C26	0.2924 (3)	0.9391 (2)	0.32750 (13)	0.0317 (5)	
H26	0.2804	0.9650	0.2799	0.038*	
C27	0.3894 (3)	0.8616 (2)	0.33856 (12)	0.0314 (5)	
H27	0.4425	0.8340	0.2987	0.038*	
C28	0.4054 (2)	0.82602 (19)	0.41109 (12)	0.0268 (4)	
C29	0.0727 (2)	0.65426 (19)	1.07554 (12)	0.0275 (4)	
C30	0.0789 (3)	0.6144 (2)	1.14621 (13)	0.0319 (5)	
H30	0.0020	0.6224	1.1781	0.038*	
C31	0.2025 (3)	0.5627 (2)	1.16787 (13)	0.0326 (5)	
H31	0.2091	0.5345	1.2147	0.039*	
C32	0.3170 (3)	0.55263 (19)	1.11963 (12)	0.0288 (4)	
C33	0.3143 (2)	0.59188 (18)	1.04918 (11)	0.0260 (4)	
H33	0.3921	0.5835	1.0179	0.031*	
C34	0.1905 (2)	0.64437 (18)	1.02687 (12)	0.0250 (4)	
C35	−0.0731 (3)	0.7797 (2)	0.91302 (14)	0.0330 (5)	
H35	−0.1658	0.8052	0.9203	0.040*	
C36	−0.0061 (3)	0.7904 (2)	0.84590 (13)	0.0315 (4)	
H36	−0.0567	0.8226	0.8073	0.038*	
C37	0.4842 (3)	0.6770 (2)	0.63531 (13)	0.0309 (4)	
H37	0.5258	0.6340	0.6696	0.037*	
C38	0.5315 (3)	0.6729 (2)	0.56168 (13)	0.0315 (5)	
H38	0.6027	0.6278	0.5463	0.038*	

### Atomic displacement parameters (Å^2^)

**Table:**

	*U*^11^	*U*^22^	*U*^33^	*U*^12^	*U*^13^	*U*^23^
Br1	0.03362 (13)	0.04857 (15)	0.03772 (14)	0.02071 (10)	0.00922 (10)	0.01996 (10)
Br2	0.03889 (14)	0.04713 (15)	0.03280 (14)	0.02262 (11)	0.01095 (10)	0.01533 (10)
N1	0.0315 (9)	0.0318 (9)	0.0308 (9)	0.0152 (7)	0.0158 (8)	0.0076 (7)
N2	0.0274 (9)	0.0350 (9)	0.0298 (9)	0.0131 (7)	0.0133 (7)	0.0092 (7)
C1	0.0410 (14)	0.0496 (15)	0.0563 (16)	0.0221 (12)	0.0036 (12)	0.0093 (12)
C2	0.0280 (10)	0.0365 (12)	0.0435 (13)	0.0109 (9)	0.0136 (10)	0.0052 (10)
C3	0.0285 (10)	0.0356 (11)	0.0324 (11)	0.0096 (9)	0.0136 (9)	0.0063 (9)
C4	0.0292 (10)	0.0352 (11)	0.0302 (11)	0.0096 (9)	0.0123 (9)	0.0063 (8)
C5	0.0341 (11)	0.0317 (11)	0.0347 (11)	0.0126 (9)	0.0154 (9)	0.0081 (8)
C6	0.0311 (11)	0.0309 (11)	0.0367 (11)	0.0143 (8)	0.0144 (9)	0.0028 (9)
C7	0.0254 (9)	0.0256 (10)	0.0281 (10)	0.0076 (8)	0.0099 (8)	0.0058 (8)
C8	0.0217 (9)	0.0237 (9)	0.0269 (10)	0.0070 (7)	0.0067 (8)	0.0051 (7)
C9	0.0257 (10)	0.0277 (10)	0.0290 (11)	0.0105 (8)	0.0109 (8)	0.0069 (8)
C10	0.0267 (10)	0.0276 (10)	0.0307 (11)	0.0097 (8)	0.0099 (8)	0.0084 (8)
C11	0.0321 (11)	0.0343 (11)	0.0289 (11)	0.0134 (9)	0.0090 (9)	0.0147 (8)
C12	0.0328 (11)	0.0314 (11)	0.0277 (11)	0.0133 (9)	0.0091 (9)	0.0114 (8)
C13	0.0304 (10)	0.0278 (10)	0.0295 (11)	0.0104 (8)	0.0110 (9)	0.0077 (8)
C14	0.0271 (10)	0.0288 (11)	0.0279 (11)	0.0116 (8)	0.0111 (8)	0.0065 (8)
C15	0.0267 (10)	0.0265 (10)	0.0268 (10)	0.0092 (8)	0.0088 (8)	0.0034 (8)
C16	0.0272 (10)	0.0284 (10)	0.0297 (10)	0.0101 (8)	0.0109 (8)	0.0053 (8)
C17	0.0252 (10)	0.0363 (11)	0.0356 (11)	0.0128 (8)	0.0137 (9)	0.0050 (9)
C18	0.0299 (11)	0.0395 (12)	0.0380 (12)	0.0144 (9)	0.0099 (9)	0.0002 (9)
C19	0.0351 (11)	0.0414 (12)	0.0303 (11)	0.0193 (10)	0.0107 (9)	0.0034 (9)
C20	0.0326 (11)	0.0320 (11)	0.0293 (11)	0.0111 (9)	0.0130 (9)	0.0052 (8)
C21	0.0311 (10)	0.0329 (11)	0.0344 (11)	0.0113 (8)	0.0129 (9)	0.0046 (8)
C22	0.0398 (13)	0.0455 (14)	0.0540 (15)	0.0190 (11)	0.0112 (11)	0.0134 (11)
C23	0.0242 (9)	0.0247 (9)	0.0227 (9)	0.0057 (7)	0.0069 (8)	0.0044 (7)
C24	0.0222 (9)	0.0257 (10)	0.0259 (10)	0.0076 (7)	0.0067 (8)	0.0054 (7)
C25	0.0242 (9)	0.0309 (10)	0.0287 (10)	0.0080 (8)	0.0068 (8)	0.0088 (8)
C26	0.0321 (11)	0.0392 (12)	0.0249 (10)	0.0085 (9)	0.0083 (9)	0.0114 (8)
C27	0.0328 (11)	0.0363 (11)	0.0261 (10)	0.0099 (9)	0.0125 (9)	0.0059 (8)
C28	0.0260 (10)	0.0278 (10)	0.0268 (10)	0.0079 (8)	0.0100 (8)	0.0038 (8)
C29	0.0269 (10)	0.0272 (10)	0.0282 (10)	0.0084 (8)	0.0103 (8)	0.0024 (8)
C30	0.0338 (11)	0.0364 (11)	0.0280 (11)	0.0108 (9)	0.0154 (9)	0.0064 (8)
C31	0.0386 (12)	0.0363 (11)	0.0262 (10)	0.0124 (9)	0.0120 (9)	0.0096 (8)
C32	0.0310 (10)	0.0302 (11)	0.0265 (10)	0.0116 (8)	0.0090 (8)	0.0042 (8)
C33	0.0274 (10)	0.0272 (10)	0.0244 (10)	0.0096 (8)	0.0094 (8)	0.0036 (7)
C34	0.0261 (10)	0.0248 (10)	0.0239 (10)	0.0073 (8)	0.0082 (8)	0.0032 (7)
C35	0.0298 (11)	0.0345 (11)	0.0415 (12)	0.0163 (9)	0.0138 (9)	0.0112 (9)
C36	0.0315 (11)	0.0341 (11)	0.0349 (11)	0.0155 (9)	0.0098 (9)	0.0126 (9)
C37	0.0307 (10)	0.0323 (11)	0.0360 (12)	0.0148 (8)	0.0099 (9)	0.0133 (9)
C38	0.0309 (10)	0.0324 (11)	0.0393 (12)	0.0182 (9)	0.0140 (9)	0.0106 (9)

### Geometric parameters (Å, º)

**Table:**

Br1—C25	1.906 (2)	C16—C35	1.388 (3)
Br2—C32	1.907 (2)	C17—C18	1.525 (3)
N1—C7	1.382 (3)	C17—H17A	0.9700
N1—C28	1.382 (3)	C17—H17B	0.9700
N1—C6	1.451 (2)	C18—C19	1.524 (3)
N2—C16	1.382 (3)	C18—H18A	0.9700
N2—C29	1.383 (3)	C18—H18B	0.9700
N2—C17	1.450 (2)	C19—C20	1.522 (3)
C1—C2	1.507 (3)	C19—H19A	0.9700
C1—H1A	0.9600	C19—H19B	0.9700
C1—H1B	0.9600	C20—C21	1.518 (3)
C1—H1C	0.9600	C20—H20A	0.9700
C2—C3	1.517 (3)	C20—H20B	0.9700
C2—H2A	0.9700	C21—C22	1.506 (3)
C2—H2B	0.9700	C21—H21A	0.9700
C3—C4	1.523 (3)	C21—H21B	0.9700
C3—H3A	0.9700	C22—H22A	0.9600
C3—H3B	0.9700	C22—H22B	0.9600
C4—C5	1.519 (3)	C22—H22C	0.9600
C4—H4A	0.9700	C23—C24	1.389 (3)
C4—H4B	0.9700	C23—C28	1.412 (3)
C5—C6	1.524 (3)	C24—C25	1.381 (3)
C5—H5A	0.9700	C24—H24	0.9300
C5—H5B	0.9700	C25—C26	1.392 (3)
C6—H6A	0.9700	C26—C27	1.383 (3)
C6—H6B	0.9700	C26—H26	0.9300
C7—C38	1.388 (3)	C27—C28	1.394 (3)
C7—C8	1.411 (3)	C27—H27	0.9300
C8—C9	1.391 (3)	C29—C30	1.386 (3)
C8—C23	1.438 (3)	C29—C34	1.417 (3)
C9—C10	1.393 (3)	C30—C31	1.380 (3)
C9—H9	0.9300	C30—H30	0.9300
C10—C37	1.412 (3)	C31—C32	1.390 (3)
C10—C11	1.466 (3)	C31—H31	0.9300
C11—C12	1.329 (3)	C32—C33	1.380 (3)
C11—H11	0.9300	C33—C34	1.390 (3)
C12—C13	1.469 (3)	C33—H33	0.9300
C12—H12	0.9300	C35—C36	1.382 (3)
C13—C14	1.399 (3)	C35—H35	0.9300
C13—C36	1.411 (3)	C36—H36	0.9300
C14—C15	1.387 (3)	C37—C38	1.390 (3)
C14—H14	0.9300	C37—H37	0.9300
C15—C16	1.417 (3)	C38—H38	0.9300
C15—C34	1.438 (3)		
			
C7—N1—C28	108.54 (16)	C19—C18—C17	112.46 (18)
C7—N1—C6	125.99 (18)	C19—C18—H18A	109.1
C28—N1—C6	125.38 (18)	C17—C18—H18A	109.1
C16—N2—C29	108.88 (16)	C19—C18—H18B	109.1
C16—N2—C17	126.08 (18)	C17—C18—H18B	109.1
C29—N2—C17	125.02 (18)	H18A—C18—H18B	107.8
C2—C1—H1A	109.5	C20—C19—C18	114.68 (18)
C2—C1—H1B	109.5	C20—C19—H19A	108.6
H1A—C1—H1B	109.5	C18—C19—H19A	108.6
C2—C1—H1C	109.5	C20—C19—H19B	108.6
H1A—C1—H1C	109.5	C18—C19—H19B	108.6
H1B—C1—H1C	109.5	H19A—C19—H19B	107.6
C1—C2—C3	113.9 (2)	C21—C20—C19	112.96 (17)
C1—C2—H2A	108.8	C21—C20—H20A	109.0
C3—C2—H2A	108.8	C19—C20—H20A	109.0
C1—C2—H2B	108.8	C21—C20—H20B	109.0
C3—C2—H2B	108.8	C19—C20—H20B	109.0
H2A—C2—H2B	107.7	H20A—C20—H20B	107.8
C2—C3—C4	113.19 (18)	C20—C21—C22	114.16 (19)
C2—C3—H3A	108.9	C20—C21—H21A	108.7
C4—C3—H3A	108.9	C22—C21—H21A	108.7
C2—C3—H3B	108.9	C20—C21—H21B	108.7
C4—C3—H3B	108.9	C22—C21—H21B	108.7
H3A—C3—H3B	107.8	H21A—C21—H21B	107.6
C3—C4—C5	114.11 (17)	C21—C22—H22A	109.5
C3—C4—H4A	108.7	C21—C22—H22B	109.5
C5—C4—H4A	108.7	H22A—C22—H22B	109.5
C3—C4—H4B	108.7	C21—C22—H22C	109.5
C5—C4—H4B	108.7	H22A—C22—H22C	109.5
H4A—C4—H4B	107.6	H22B—C22—H22C	109.5
C4—C5—C6	112.79 (17)	C24—C23—C28	119.99 (18)
C4—C5—H5A	109.0	C24—C23—C8	133.57 (17)
C6—C5—H5A	109.0	C28—C23—C8	106.41 (17)
C4—C5—H5B	109.0	C25—C24—C23	117.61 (17)
C6—C5—H5B	109.0	C25—C24—H24	121.2
H5A—C5—H5B	107.8	C23—C24—H24	121.2
N1—C6—C5	113.69 (17)	C24—C25—C26	122.94 (19)
N1—C6—H6A	108.8	C24—C25—Br1	118.72 (15)
C5—C6—H6A	108.8	C26—C25—Br1	118.33 (16)
N1—C6—H6B	108.8	C27—C26—C25	119.9 (2)
C5—C6—H6B	108.8	C27—C26—H26	120.1
H6A—C6—H6B	107.7	C25—C26—H26	120.1
N1—C7—C38	129.56 (18)	C28—C27—C26	118.18 (18)
N1—C7—C8	109.08 (17)	C28—C27—H27	120.9
C38—C7—C8	121.37 (18)	C26—C27—H27	120.9
C9—C8—C7	120.07 (18)	N1—C28—C27	129.37 (18)
C9—C8—C23	133.19 (18)	N1—C28—C23	109.24 (18)
C7—C8—C23	106.73 (16)	C27—C28—C23	121.38 (19)
C8—C9—C10	119.78 (17)	C30—C29—N2	129.50 (18)
C8—C9—H9	120.1	C30—C29—C34	121.60 (19)
C10—C9—H9	120.1	N2—C29—C34	108.90 (18)
C9—C10—C37	118.72 (18)	C29—C30—C31	118.11 (18)
C9—C10—C11	122.78 (17)	C29—C30—H30	120.9
C37—C10—C11	118.46 (19)	C31—C30—H30	120.9
C12—C11—C10	126.0 (2)	C30—C31—C32	120.0 (2)
C12—C11—H11	117.0	C30—C31—H31	120.0
C10—C11—H11	117.0	C32—C31—H31	120.0
C11—C12—C13	127.3 (2)	C33—C32—C31	123.2 (2)
C11—C12—H12	116.4	C33—C32—Br2	118.65 (15)
C13—C12—H12	116.4	C31—C32—Br2	118.17 (16)
C14—C13—C36	118.61 (18)	C32—C33—C34	117.33 (18)
C14—C13—C12	122.78 (18)	C32—C33—H33	121.3
C36—C13—C12	118.61 (19)	C34—C33—H33	121.3
C15—C14—C13	119.81 (17)	C33—C34—C29	119.81 (19)
C15—C14—H14	120.1	C33—C34—C15	133.55 (18)
C13—C14—H14	120.1	C29—C34—C15	106.61 (17)
C14—C15—C16	119.93 (19)	C16—C35—C36	117.65 (19)
C14—C15—C34	133.42 (18)	C16—C35—H35	121.2
C16—C15—C34	106.66 (17)	C36—C35—H35	121.2
N2—C16—C35	129.78 (18)	C35—C36—C13	122.7 (2)
N2—C16—C15	108.96 (18)	C35—C36—H36	118.6
C35—C16—C15	121.26 (18)	C13—C36—H36	118.6
N2—C17—C18	113.14 (17)	C38—C37—C10	122.59 (19)
N2—C17—H17A	109.0	C38—C37—H37	118.7
C18—C17—H17A	109.0	C10—C37—H37	118.7
N2—C17—H17B	109.0	C7—C38—C37	117.42 (18)
C18—C17—H17B	109.0	C7—C38—H38	121.3
H17A—C17—H17B	107.8	C37—C38—H38	121.3

### Hydrogen-bond geometry (Å, º)

Cg6 is the centroid of the C29–C34 ring.

**Table:**

*D*—H···*A*	*D*—H	H···*A*	*D*···*A*	*D*—H···*A*
C9—H9···Br1^i^	0.93	3.04	3.9062 (19)	157
C12—H12···Br1^i^	0.93	3.00	3.921 (2)	172
C11—H11···Br2^ii^	0.93	3.03	3.932 (2)	163
C14—H14···Br2^ii^	0.93	2.94	3.821 (2)	159
C21—H21*B*···*Cg*6^iii^	0.93	2.89	3.791 (3)	154

Symmetry codes: (i) −*x*, −*y*+2, −*z*+1; (ii) −*x*+1, −*y*+1, −*z*+2; (iii) *x*−1, *y*, *z*−1.

### Analysis of Short Ring-Interactions with Cg-Cg (Å, °)

**Table:**

Cg(I)	Cg(J) [ ARU(J)]	Cg-Cg	α	CgI_Perp	CgJ_Perp
Cg(1)	Cg(1) [2656.01]	4.3365	0	-3.5042	-3.5042
Cg(1)	Cg(3) [2656.01]	5.4195	1.424	-3.5070	-3.5193
Cg(1)	Cg(5) [2656.01]	3.6898	1.126	-3.5290	-3.5284
Cg(2)	Cg(2) [2765.01]	4.0065	0	3.5166	3.5166
Cg(2)	Cg(4) [2765.01]	5.2650	0.997	3.5672	3.5002
Cg(2)	Cg(6) [2765.01]	3.5000	1.275	3.4956	3.4909
Cg(3)	Cg(1) [2656.01]	5.4195	1.424	-3.5193	-3.5070
Cg(3)	Cg(5) [2656.01]	4.0524	1.960	-3.5814	-3.5354
Cg(4)	Cg(2) [2765.01]	5.2650	0.997	3.5002	3.5672
Cg(4)	Cg(6) [2765.01]	4.0191	2.123	3.4465	3.5085
Cg(5)	Cg(1) [2656.01]	3.6898	1.126	-3.5284	-3.5290
Cg(5)	Cg(3) [2656.01]	4.0524	1.960	-3.5354	-3.5814
Cg(5)	Cg(5) [2656.01]	4.2114	0	-3.5109	-3.5109
Cg(5)	Cg(6) [1556.01]	5.4638	16.970	-4.0104	3.0948
Cg(6)	Cg(2) [2765.01]	3.5000	1.275	3.4909	3.4956
Cg(6)	Cg(4) [2765.01]	4.0191	2.123	3.5085	3.4465
Cg(6)	Cg(5) [1554.01]	5.4638	16.970	3.0948	-4.0104
Cg(6)	Cg(6) [2765.01]	4.2119	0	3.5165	3.5165

Cg(I), Cg(J): Plane number I,J (ring number in figure 1); Cg-Cg: Distance between ring Centroids (Ang.); α: Dihedral Angle between Planes I and J (Deg); CgI_Perp: Perpendicular distance of Cg(I) on ring J (Ang.); CgJ_Perp: Perpendicular distance of Cg(J) on ring I (Ang.); [2656.01]: 1-X, -Y, 1-Z; [2765.01] = 2-X, 1-Y, -Z; [1556.01] = X, Y, 1+Z; [1554.01] = X, Y, -1+Z.

### The fractional coordinates of single crystal

**Table:**

*CgI*	*x*	*y*	*z*
*Cg*1	0.589228	0.201962	0.526550
*Cg*2	0.925482	0.314678	-0.014062
*Cg*3	0.576427	0.257533	0.414090
*Cg*4	0.930362	0.257838	0.097958
*Cg*5	0.691673	0.097473	0.600602
*Cg*6	0.804030	0.396628	-0.097550
